# Affective resonance and place bonds: the roles of empathy and place attachment for pro-environmental behavior

**DOI:** 10.3389/fpsyg.2026.1820194

**Published:** 2026-05-26

**Authors:** Wanxia Jiang

**Affiliations:** School of Housing, Building and Planning, Universiti Sains Malaysia, Penang, Malaysia

**Keywords:** human–environment interaction, landscape perception, multidimensional empathy, place attachment, pro-environmental behavior

## Abstract

As environmental pollution intensifies and ecological challenges become more pronounced, fostering pro-environmental behavior (PEB) is increasingly important for sustainable urban development. Although prior studies have linked environmental perception to environmental behavior, the emotional mechanisms underlying this relationship remain insufficiently explored, especially the joint roles of multiple pathways. This study examines how landscape perception is associated with pro-environmental behavior through empathy and place attachment. A questionnaire survey was conducted with 478 users across three comprehensive urban parks in Chengdu, China. Structural equation modeling was applied to estimate direct and indirect relationships among landscape perception, emotional responses, and PEB. Results indicate that positive landscape perceptions significantly promote PEB both directly and indirectly. Empathy and place attachment serve as key mediators, with empathy exerting a stronger mediating effect. Clarifying these differentiated emotional pathways advances understanding of environment and behavior relationships and informs the design and management of urban green spaces to support broader urban environmental governance.

## Highlights

- This study conceptualizes landscape perception as a multidimensional construct that incorporates both physical and psychological dimensions of experience, reflecting the growing attention to psychological needs and supporting human wellbeing.
- This study extends empathy from its psychological roots to the context of urban public spaces, establishing a new emotional pathway for understanding user–landscape interactions.
- This study extends empathy beyond natural empathy to encompass spatial empathy in urban parks.
- This study addresses an overlooked gap by testing the relationship between natural empathy and place attachment, providing the first empirical support for this linkage.
- This study demonstrates that empathy and place attachment jointly mediate the influence of landscape perception on pro-environmental behavior, with empathy exhibiting a stronger mediating effect.

## 1 Introduction

With the continued advancement of urbanization, environmental pollution and ecological pressure in urban areas have become increasingly pronounced. How to achieve sustainable management of public spaces under conditions of intensive use has therefore emerged as a key concern in urban planning and landscape research (Bianconi et al., 2023; Manisalidis et al., 2020). As the most accessible and frequently used type of public green space, urban parks not only provide important functions related to ecological regulation, environmental improvement, and biodiversity conservation, but are also deeply embedded in residents' daily practices through repeated use, activities, and social interactions (Konijnendijk et al., 2013; Wang et al., 2024). In this context, the maintenance of environmental quality and ecological protection in urban parks largely depends on users' behavioral patterns and levels of participation, such as whether vegetation is respected, environmental cleanliness is maintained, and protective attitudes toward public spaces are expressed (Ayala-Azcárraga et al., 2019).

However, existing studies consistently indicate that environmentally detrimental behaviors, including vegetation damage, littering, and neglect of environmental maintenance, remain widespread in urban parks and other public spaces (Colding et al., 2020; Zhang et al., 2024). This persistence indicates that reliance on institutional regulation or governance technologies alone is insufficient to fundamentally alleviate environmental pressure in public spaces. Compared with *post hoc* management, understanding why and how individuals adopt pro-environmental behaviors at the stage of behavioral formation has been recognized as a more effective pathway for improving environmental quality and achieving the long-term sustainable management of urban parks (Kruize et al., 2019). Consequently, understanding how individuals translate their perceptions and evaluations of environmental value into pro-environmental intentions and behaviors has become a critical issue in urban landscape research. This raises a further question: why does environmental awareness not consistently translate into pro-environmental behavior in urban park settings?

Previous studies have attempted to address this issue from different perspectives. A substantial body of research has identified place attachment as a key predictor of pro-environmental behavior, suggesting that individuals who develop strong emotional bonds with a place are more likely to engage in protective actions (Bošnjaković and Radionov, 2018; Scannell and Gifford, 2017). In parallel, other studies have highlighted the role of empathy-related processes in shaping environmental concern and behavioral intention (Chen et al., 2024; Ienna et al., 2022). In addition, research on environmental perception has demonstrated that individuals' evaluations of landscape qualities can influence behavioral responses in public spaces (Genzer et al., 2023; Pavlovich and Krahnke, 2012).

Despite extensive research on pro-environmental behavior from various perspectives, existing studies have not yet provided a fully integrated explanation of how pro-environmental behavior are formed in urban park contexts. Previous research has examined the roles of place attachment, empathy, and environmental perception in shaping environmental behavior, often focusing on their direct relationships with behavioral outcomes, while paying less attention to how these factors interact within the process of environmental experience (Brown et al., 2019; Halpenny, 2010; Junot et al., 2018). Meanwhile, studies on environmental or landscape perception have largely concentrated on preference, evaluation, or restorative outcomes, rather than explaining how perceived landscape characteristics are translated into pro-environmental intentions through affective mechanisms (DeVille et al., 2021; Li X. et al., 2023). Within this line of research, place attachment is typically treated as a relatively stable antecedent of pro-environmental behavior (Chen et al., 2024; Halpenny, 2010; Xu and Han, 2019), with limited attention given to the possibility that it may itself be shaped by prior affective responses during environmental experience. More importantly, empathy with nature has been widely recognized as an important emotional mechanism in human–environment relationships (Li et al., 2024; Wang et al., 2023). However, the pathway through which empathy contributes to the formation of place attachment remains underexplored. Furthermore, although emotional responses have long been acknowledged as crucial in shaping human–environment relationships, they are often treated as post-experience outcomes rather than being incorporated into the process of behavioral formation, making it difficult to explain how emotional processes are activated during landscape experience and subsequently contribute to pro-environmental behavior (Kellert, 2012; Schauer et al., 2016; Zhang et al., 2018). Specifically, although prior studies have identified place attachment, empathy, and environmental perception as important predictors of pro-environmental behavior, it remains unclear how these factors are sequentially connected within the process of environmental experience to shape pro-environmental behavior in urban park contexts. In particular, the potential role of empathy as a preceding affective mechanism that contributes to the formation of place attachment has not been sufficiently clarified, highlighting a critical gap in understanding the affective pathways underlying pro-environmental behavior.

To address these gaps, this study proposes a process-oriented framework that links landscape perception, empathy, and place attachment within the stimulus–organism–response perspective. This study aims to examine how landscape perception is associated with pro-environmental behavior through a sequential affective process involving empathy and place attachment. Rather than treating place attachment as a given antecedent, this study conceptualizes it as an outcome shaped by preceding emotional responses, and examines how empathy functions as a key affective mechanism that plays a role in the formation of place attachment and is associated with pro-environmental behavior. This perspective differs from previous research that primarily considers place attachment or empathy as independent predictors, by explicitly modeling their sequential relationship within environmental experience. Importantly, this study further distinguishes between empathy and place attachment as affective mechanisms operating at different temporal scales, capturing immediate emotional responses and more stable place-based bonds, respectively. By integrating these differentiated emotional processes with environmental perception, the study provides a more comprehensive explanation of how pro-environmental behavior are formed in urban park contexts. The findings contribute to a process-based understanding of human–environment relationships and offer practical implications for the design and management of urban parks to encourage pro-environmental behavior.

## 2 Literature review

### 2.1 Theoretical foundation

The Stimulus–Organism–Response (SOR) framework offers a well-established theoretical lens for understanding how environmental stimuli are associated with behavioral outcomes through internal psychological processes (Mehrabian and Russell, 1974). Rather than assuming a direct link between environment and behavior, SOR emphasizes the mediating role of individuals' affective and cognitive states in shaping responses to external settings (Yin et al., 2023). This perspective is particularly relevant to urban parks, where environmental experiences are often accompanied by complex emotional processes and behavioral choices.

In recent years, the SOR framework has been increasingly adopted in landscape and park studies to explain individuals' psychological responses and behavioral tendencies within specific spatial contexts. Prior research has examined, for example, the role of social norms in littering behavior in urban parks (Fenitra et al., 2023), as well as the relationship between restorative environmental perceptions and pro-environmental behavior through place attachment (Xu et al., 2022). Nevertheless, existing applications of the SOR framework in this field have largely focused on functional or single-dimensional perceptual factors, offering limited insight into the emotional processes linking landscape experiences with pro-environmental behavior in public spaces.

Within urban parks, which encompass both natural and cultural dimensions, visitors' responses extend beyond immediate actions to include emotional resonance and deeper affective bonds with place (Kong et al., 2022). Building on this insight, the present study applies the SOR framework by incorporating empathy and place attachment as organismic variables operating at different temporal scales, and by integrating multidimensional landscape perception with pro-environmental behavior. To address this limitation, the present study applies the SOR framework by incorporating empathy and place attachment as organismic variables operating at different temporal scales, and by integrating multidimensional landscape perception with pro-environmental behavior.

### 2.2 Hypothesis development

#### 2.2.1 Landscape perception and emotional bonds

Landscape perception in human-environment interaction shapes external cognitive impressions of the environment while simultaneously stimulating emotional responses through multisensory experiences (Dong et al., 2024). Landscape perception extends beyond cognitive appraisal to encompass the integrated interpretation of sensory stimuli, spatial qualities, and environmental meaning, thereby activating affective responses beyond general emotional states. These affective outcomes are often described as emotional bonds, in which empathy reflects immediate resonance with the environment, whereas place attachment develops through longer-term interaction and identification (Diener and Hagen, 2022; Li J. et al., 2023). Visual and auditory features such as greenery, spatial form, and natural sounds have been shown to evoke positive emotions and restoration (Gan et al., 2022; Shabani et al., 2020). Integrating multiple sensory cues further enhances wellbeing beyond the effects of single dimensions (Zhang et al., 2019).

However, emotional responses to landscape are not limited to general states such as pleasure or restoration. When individuals perceive environmental characteristics as meaningful, harmonious, or engaging, these perceptions may foster a sense of affective resonance with natural or spatial elements, which is conceptually aligned with empathy as an immediate emotional response to external stimuli (DeVille et al., 2021). Previous studies have also suggested that meaningful environmental experiences and emotional engagement can enhance individuals' sensitivity and responsiveness to their surroundings (Schreuder et al., 2016), providing indirect support for the linkage between perception and empathy. Therefore, this study conceptualizes landscape perception as a composite stimulus that fosters empathetic responses.

Landscape perception also provides the basis for place attachment, a sense of belonging and identification that extends beyond functional dependence (Fagerholm et al., 2020; Liu et al., 2020). Environmental quality, activity opportunities, and perceived naturalness have been linked to stronger attachment in parks (Neuvonen et al., 2010; Wartmann et al., 2021b). Place attachment does not arise solely from physical attributes of a place, but from how these attributes are perceived, emotionally experienced, and gradually associated with personal meaning (Hashemnezhad et al., 2013). However, existing work emphasizes physical attributes while overlooking the affective experiences through which landscapes gradually become meaningful places (Urry, 2016). More favorable landscape perception may not only enhance immediate emotional responses but also strengthen the development of place attachment by reinforcing individuals' sense of belonging, identification, and emotional connection to the environment (Bow and Buys, 2003). Accordingly, this study advances the following hypotheses:

**H1:** Landscape perception has a positive effect on empathy.
**H2:** Landscape perception has a positive effect on place attachment.

#### 2.2.2 Empathy and place attachment

Empathy is an emotional response that emerges from perceiving external environments or others' feelings, and it strengthens affective resonance between people and their surroundings (Pavlovich and Krahnke, 2012). Social psychology highlights its role in fostering understanding and responsibility, which extends to human–nature relationships where empathy is associated with pro-environmental attitudes and behaviors (Ienna et al., 2022; Lengieza et al., 2023). Within urban parks, empathic responses to natural elements or spatial atmospheres can enhance individuals' emotional engagement with their surroundings. Place attachment, in contrast, refers to a relatively stable emotional bond with a place, which develops through repeated experience, personal meaning, and a growing sense of belonging and identification (Halpenny, 2010; Rapuano et al., 2022). Previous studies have shown that affective experiences such as pleasure, wellbeing, and emotional engagement are closely related to the development of place attachment, as they reinforce the personal significance of a place and strengthen emotional identification with it (Reitsamer and Brunner-Sperdin, 2017; Trinanda et al., 2022). From this theoretical standpoint, empathy may be regarded as an earlier affective mechanism in the formation of place attachment. This is because empathy involves affective resonance with environmental characteristics, whereas place attachment reflects a more stable bond that emerges when emotional experiences become internalized as place-based meaning, belonging, and identification (Halpenny, 2010; Pavlovich and Krahnke, 2012). In this sense, empathic engagement with natural or spatial elements may deepen individuals' emotional connection to a place and facilitate the gradual transformation of immediate affective responses into more enduring place attachment (Brown et al., 2019). These findings provide a theoretical basis for proposing that empathy, as a form of affective resonance, is positively associated with place attachment. Hence, this study proposes the following hypothesis:

**H3:** Empathy has a positive effect on place attachment.

#### 2.2.3 Emotional bonds and pro-environmental behavior

Research has shown that individuals' emotional responses are a crucial psychological factor associated with pro-environmental behavior (Tian and Liu, 2022). When confronted with signs of environmental degradation in parks, such as polluted water, accumulated litter, or damaged vegetation, people often feel regret, concern, or moral unease, which may strengthen their sense of responsibility toward nature and be related to protective actions (Clayton and Myers, 2015; Cooper and Palmer, 2005). Studies further indicate that individuals with stronger connectedness to nature exhibit higher levels of environmental empathy and are more likely to engage in practices such as waste sorting and resource conservation (Restall and Conrad, 2015; Teixeira et al., 2023). These findings suggest that empathy can be understood as both an emotional experience and a psychological factor linked to pro-environmental behavior.

Place attachment reflects individuals' sense of belonging and identification with a specific place, playing a central role in explaining pro-environmental actions by linking environmental quality with their emotional identity (Daryanto and Song, 2021). Empirical research indicates that visitors with stronger attachment to parks or recreational sites are more likely to protect ecological quality through actions such as picking up litter, complying with park regulations, or supporting sustainable management (Esfandiar et al., 2020). This attachment develops through repeated experiences and accumulated emotions, through which individuals may come to perceive protecting a place as safeguarding both self and home (Koupatsiaris and Drinia, 2024).

In addition, landscape perception may be associated with individuals' pro-environmental tendencies, as positive perceptions of naturalness and aesthetic qualities are related to favorable environmental attitudes and protective actions (Duradoni et al., 2025; Zhang et al., 2023). Taken together, these findings indicate that both landscape perception and emotional bonds are closely related to pro-environmental behavior. Accordingly, this study proposes the following hypotheses:

**H4:** Empathy has a positive effect on pro-environmental behavior.
**H5:** Place attachment has a positive effect on pro-environmental behavior.
**H6:** Landscape perception has a positive effect on pro-environmental behavior.

**Figure 1:**
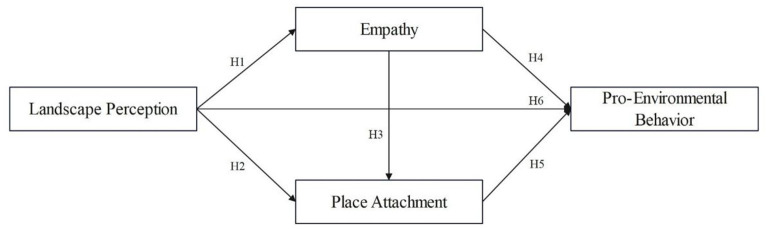
The conceptual model.

Based on the above analysis, the study constructs a conceptual framework to illustrate the relationships among landscape perception, emotional bonds (empathy and place attachment), and pro-environmental behavior. The framework is grounded in the SOR theory, highlighting how external landscape stimuli are associated with emotional mechanisms and behavioral responses. Figure 1 presents the hypothesized structural paths, including the relationships between landscape perception, emotional bonds, and pro-environmental behavior, as well as the mediating roles of empathy and place attachment.

## 3 Methods

### 3.1 Study site and participants

**Figure 2:**
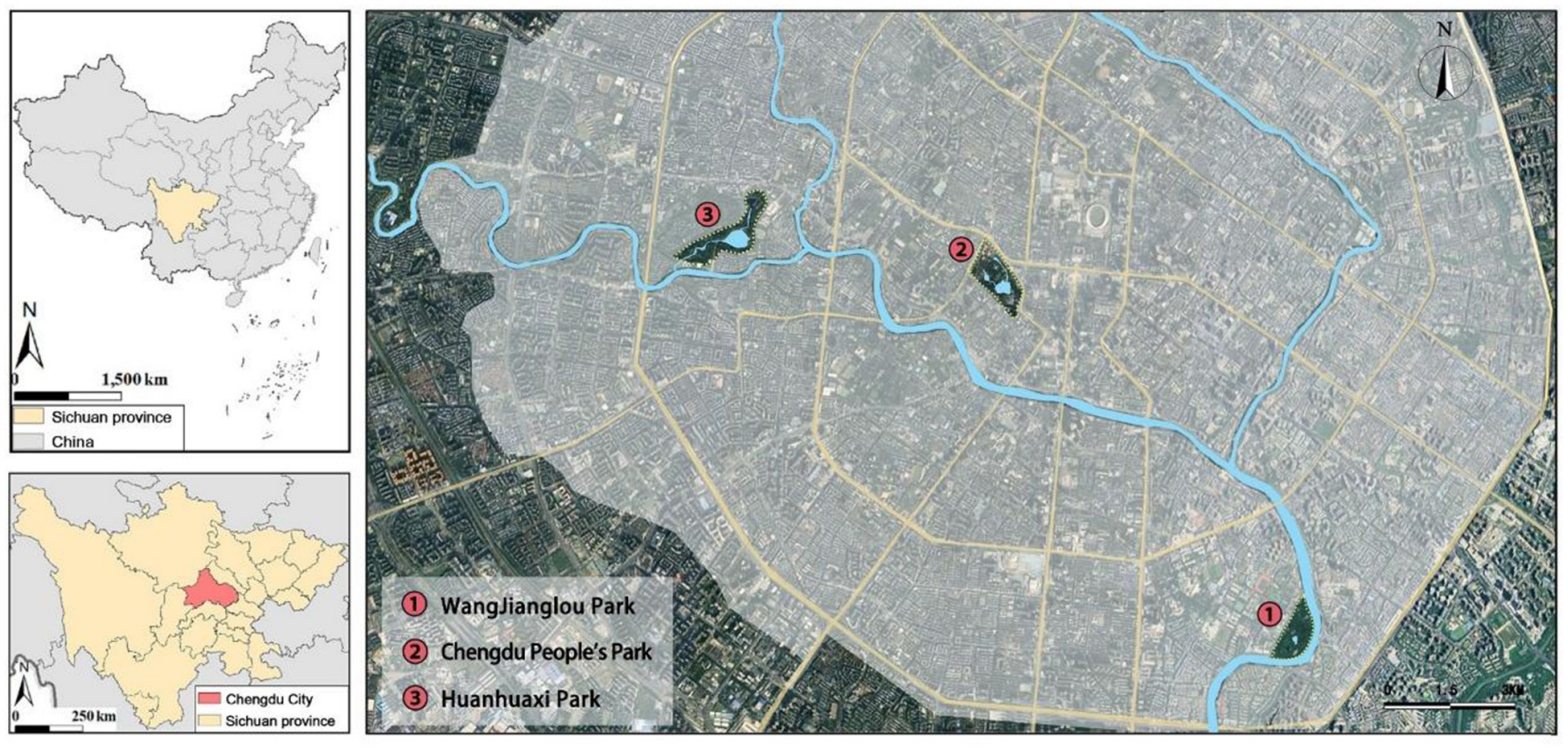
Geographic location of the three sample parks in Chengdu City, China. *Source:* Adapted from Google maps (2026).

The study was conducted in Chengdu, a major metropolitan center in southwest China and the capital of Sichuan Province. As one of China's rapidly urbanizing cities, Chengdu has experienced increasing environmental pressures, including urban pollution, high visitor density in public spaces, and growing demand for sustainable park management. In response, the city has actively promoted the “Park City” initiative, aiming to improve ecological quality and encourage pro-environmental behavior among residents. This context provides a relevant setting for examining how urban park environments are associated with individuals' environmental perceptions and behavioral responses. Among the city's numerous urban parks, Wangjianglou Park, Chengdu People's Park, and Huanhuaxi Park were selected as survey sites. All three parks are located within the urban core, exhibit high public accessibility and frequent daily use, and represent distinct spatial forms, cultural attributes, and functional characteristics. The spatial distribution of the sample parks is illustrated in Figure 2.

Participants in this study were adult visitors to these parks, as park users are directly exposed to landscape environments and are more likely to develop immediate perceptions, emotional responses, and pro-environmental behavior during their visits. To ensure the relevance and reliability of responses, only individuals aged 18 and above who were actively using the park spaces during the survey period were invited to participate. This sampling approach allows for capturing real-time environmental experiences and their associations with pro-environmental behavior.

### 3.2 Sample characteristics

Table 1 presents the socio-demographic characteristics of the sample. Among the 478 valid respondents, the gender distribution was relatively balanced, with 48.5% male and 51.5% female. The overall age structure indicated that respondents were distributed across all age groups, with a relatively higher proportion of middle-aged and older individuals, suggesting that the sample includes participants with varied life experiences. In terms of education, more than half of the participants held an associate or bachelor's degree (53.3%), while a smaller share possessed a master's degree or above, indicating a moderate to relatively high educational level within the sample. With respect to occupation, corporate employees and retirees were the dominant groups, reflecting a mix of working and non-working populations. Household income was concentrated between Chinese Yuan (CNY) 2,001 and 10,000, representing over 70% of the sample, which corresponds to the typical income range of urban residents in the study context. Overall, the sample demonstrates a diverse distribution across gender, age, education, occupation, and income groups. Given that the data were collected from active users in multiple urban parks with varied spatial, functional, and cultural characteristics, the sample can be considered reasonably representative of urban park users within the study context, as it captures a broad range of user characteristics and park-use experiences.

**Table 1:** Descriptive statistics of study participants (*n* = 478).

Characteristics	Categories	Frequency	Percent (%)
Gender	Male	232	48.5
	Female	246	51.5
Age	18–29 years	76	15.9
	30–39 years	125	26.2
	40–49 years	102	21.3
	50–59 years	89	18.6
	60 and above	86	18.0
Education	Middle school or below	63	13.2
	High school or vocational school	88	18.4
	Associate or bachelor's degree	255	53.3
	Master degree or above	72	15.1
Occupation	Self-employed	60	12.6
	Corporate employee	174	36.4
	Public institution employee	47	9.8
	Retiree	108	22.6
	University student	44	9.2
	Other	48	7.6
Income	CNY 2,000 or below	77	16.1
	CNY 2,001–6,000	186	38.9
	CNY 6,001–10,000	155	32.4
	CNY 10,001 or above	60	12.6

### 3.3 Questionnaire design

This study employed a structured questionnaire consisting of two main sections. The first section collected respondents' socio-demographic information, including gender, age, education level, occupation, and household income, to describe the basic characteristics and structure of the sample. The second section measured the core constructs of the proposed research framework, namely landscape perception, empathy, place attachment, and pro-environmental behavior. All measurement items were adapted from established instruments in environmental psychology and landscape research and were revised appropriately to fit the urban park context of this study.

Landscape perception was conceptualized as a multidimensional construct reflecting individuals' overall perceptual experience of urban park environments. Following previous studies on environmental perception and landscape experience, this construct was operationalized through three dimensions: sensory perception, aesthetic perception, and cultural perception, which together capture both the physical qualities and symbolic meanings of park landscapes (Raymond et al., 2010). It was measured using 12 items across these three dimensions. These items were used to assess how respondents perceived and evaluated the sensory, visual, and cultural characteristics of the park environment. Empathy was measured using eight items representing two dimensions: natural empathy and spatial empathy. This dimensional structure was adopted to reflect two major sources of empathic response in urban park settings, namely emotional resonance with natural elements and emotional resonance with spatial environments. The measurement of natural empathy was primarily informed by the Environmental Empathy Scale developed by Tam (2013), while the inclusion of spatial empathy was supported by studies emphasizing emotional engagement with environmental and spatial characteristics in human–environment interaction (Pavlovich and Krahnke, 2012). Accordingly, the empathy scale in this study was used to capture respondents' affective resonance with both natural features and spatial settings within the park environment.

Place attachment was measured with eight items across two dimensions, namely place identity and place dependence. This two-dimensional structure is widely established in studies of human–place relationships and has been extensively validated in environmental psychology, recreation, and leisure research (Williams and Vaske, 2003). The scale was adapted to assess respondents' emotional identification with, and functional dependence on, the park environment. Pro-environmental behavior was assessed using nine items capturing environmental attitudes and This sampling approach allows for capturing real-time environmental experiences and their associations with pro-environmental behavior in urban park contexts. The items were adapted from Ramkissoon et al. (2013) to evaluate respondents' tendencies toward environmentally responsible behavior in relation to park use and environmental protection. All items were rated on a five-point Likert scale, ranging from 1 (strongly disagree) to 5 (strongly agree). Following Brislin's (1970) translation and back-translation procedure, the questionnaire was prepared in both Chinese and English to ensure semantic accuracy and conceptual equivalence across languages.

### 3.4 Data collection

Questionnaire surveys were conducted at three representative urban parks in Chengdu between September 5 and October 25, 2024, using an on-site paper-based questionnaire approach. A convenience sampling strategy was adopted, as the selected parks attract a diverse and stable user population engaging in routine leisure and recreational activities. This sampling approach is considered appropriate for capturing real-time environmental perceptions and behavioral responses in urban park settings. To reduce potential sampling bias associated with convenience sampling, questionnaires were distributed across different locations within each park and at different times of the day, including both weekdays and weekends. This procedure helped ensure that respondents with varied park-use patterns and backgrounds were included. The target population consisted of adult park users aged 18 years or older. All participants were informed of the research purpose prior to participation, and voluntary participation was explicitly emphasized. The survey was conducted anonymously, and no personally identifiable information was collected. All procedures followed standard ethical guidelines for social science research, and the collected data were securely stored to ensure confidentiality and data integrity. A total of 600 questionnaires were distributed across the three parks, of which 560 questionnaires were returned, yielding a response rate of 93.3%. After data screening, 478 valid responses were retained for subsequent statistical analysis, which is considered adequate for Partial Least Squares Structural Equation Modeling (PLS-SEM) analysis.

## 4 Results

### 4.1 Measurement model assessment

The measurement model was assessed for reliability and convergent validity. As shown in Table 2, all constructs exhibited Cronbach's α values above 0.70, while composite reliability (CR) ranged from 0.818 to 0.897, exceeding the recommended threshold of 0.70 proposed by Hair et al. (2019), thereby indicating satisfactory internal consistency. Regarding convergent validity, all constructs achieved average variance extracted (AVE) values greater than 0.50, consistent with the criterion suggested by Henseler et al. (2015), indicating that the latent constructs adequately capture the variance of their indicators. In addition, most outer loadings exceeded 0.70, providing further support for the convergent validity of the measurement model. Overall, these results indicate that the measurement model demonstrates acceptable levels of reliability and convergent validity, supporting its suitability for subsequent analysis.

**Table 2:** Reliability and convergent validity of the measurement model.

Construct	Dimension	Item	Outer loading	Cronbach's α	CR	AVE
Landscape perception	Aesthetic perception	AP1	0.813	0.847	0.897	0.686
		AP2	0.845			
		AP3	0.821			
		AP4	0.834			
	Sensory perception	SP1	0.804	0.802	0.871	0.628
		SP2	0.808			
		SP3	0.774			
		SP4	0.782			
	Cultural perception	CP1	0.807	0.830	0.887	0.663
		CP2	0.802			
		CP3	0.826			
		CP4	0.820			
Empathy	Natural empathy	NE1	0.761	0.729	0.831	0.552
		NE2	0.715			
		NE3	0.787			
		NE4	0.706			
	Spatial empathy	SE1	0.801	0.830	0.887	0.6622
		SE2	0.804			
		SE3	0.798			
		SE4	0.850			
Place attachment	Place dependence	PD1	0.797	0.802	0.871	0.628
		PD2	0.811			
		PD3	0.772			
		PD4	0.789			
	Place identity	PI1	0.813	0.812	0.876	0.640
		PI2	0.772			
		PI3	0.815			
		PI4	0.798			
Pro-environmental behavior	Behavior attitude	BA1	0.794	0.704	0.818	0.531
		BA2	0.800			
		BA3	0.667			
		BA4	0.640			
	Behavioral intention	BI1	0.716	0.763	0.841	0.513
		BI2	0.699			
		BI3	0.754			
		BI4	0.686			
BI5	0.726

CR, composite reliability; AVE, average variance extracted.

The discriminant validity of the measurement model was assessed using the heterotrait–monotrait (HTMT) criterion (Dirgiatmo, 2023). In general, HTMT values below 0.85 indicate satisfactory discriminant validity, while values below 0.90 are still acceptable (Franke and Sarstedt, 2019). Table 3 reports that all HTMT values among the constructs were well below the threshold of 0.85, demonstrating sufficient distinctiveness among the latent variables and confirming that the model exhibits adequate discriminant validity.

**Table 3:** Heterotrait-Monotrait Ratio (HTMT) for discriminant validity.

Construct	AP	BA	BI	CP	SE	NE	PD	PI	SP
AP									
BA	0.431								
BI	0.599	0.704							
CP	0.441	0.547	0.380						
SE	0.097	0.643	0.290	0.173					
NE	0.422	0.600	0.411	0.525	0.179				
PD	0.490	0.438	0.658	0.222	0.290	0.208			
PI	0.255	0.487	0.346	0.573	0.302	0.220	0.559		
SP	0.577	0.246	0.351	0.411	0.077	0.458	0.086	0.082	

AP, aesthetic perception; BA, behavior attitude; BI, behavioral intention; CP, cultural perception; SE, spatial empathy; NE, natural empathy; PD, place dependence; PI, place identity; SP, sensory perception.

#### 4.1.1 Common method variance (CMV)

Common method variance (CMV) was evaluated using Harman's single-factor test to examine the potential impact of measurement bias in the survey data. All measurement items were entered into an exploratory factor analysis without rotation. The results showed that the first factor accounted for 22.12% of the total variance, which is below the commonly accepted threshold of 40% (Podsakoff et al., 2003). This result indicates that common method variance is unlikely to substantially bias the measurement results.

### 4.2 Structural model assessment

The structural model was assessed by examining model fit, explanatory power, and predictive relevance following the procedures recommended in PLS-SEM (Hair et al., 2019). The standardized root mean square residual (SRMR) value was 0.061, which is below the recommended threshold of 0.08, indicating a good model fit (Henseler et al., 2016). In addition, the coefficient of determination (*R*^2^) was used to evaluate the explanatory power of the endogenous constructs. The *R*^2^ values of the endogenous constructs ranged from 0.122 to 0.478, indicating a moderate level of explanatory power overall, with stronger explanatory capacity observed for pro-environmental behavior. The predictive relevance of the model was further assessed using the *Q*^2^ statistic. The *Q*^2^ values for empathy, place attachment (PA), and pro-environmental behavior (PEB) were 0.117, 0.132, and 0.257, respectively. According to Hair et al. (2021), these results indicate that the model demonstrates satisfactory predictive relevance for the endogenous constructs.

#### 4.2.1 Path coefficients and hypothesis testing

The hypotheses were evaluated based on the estimated path coefficients and their associated confidence intervals, with the results reported in Table 4. The analysis indicates that landscape perception significantly enhances empathy (β = 0.350, *t* = 7.920, *p* < 0.001) as well as place attachment (β = 0.292, *t* = 6.506, *p* < 0.001), demonstrating that the perception of environmental features can simultaneously trigger immediate emotional responses and foster a more stable attachment to place. Moreover, empathy was found to be positively associated with place attachment (β = 0.225, *t* = 5.533, *p* < 0.001), indicating that emotional resonance may be transformed into longer-term spatial bonds. With respect to the outcome variable, empathy exerted the most decisive association (β = 0.348, *t* = 9.288, *p* < 0.001), showing that emotional resonance is closely related to pro-environmental behavior. Place attachment also had a significant effect on such intentions (β = 0.288, *t* = 7.212, *p* < 0.001), highlighting the role of place-based bonds in shaping environmental responsibility. In addition, landscape perception had a significant direct effect on pro-environmental behavior (β = 0.282, *t* = 8.221, *p* < 0.001), indicating that environmental perception is associated with protective intentions. Taken together, these findings confirm that landscape perception, empathy, and place attachment are significantly associated within the model. All hypotheses (H1–H6) were supported, and the overall structural model was validated. In addition, effect sizes (*f*^2^) were calculated to assess the contribution of each exogenous construct. The *f*^2^ values ranged from 0.054 to 0.195, indicating small to moderate effect sizes according to established criteria (Cohen, 2013; Hair et al., 2019).

**Table 4:** Path coefficients and hypothesis testing results.

Hypothesis	Path	β (O)	M	STDEV	*t*-value	2.5%	95%
H1	LP → Empathy	0.350	0.351	0.044	7.920	0.250	0.428
H2	LP → PA	0.292	0.293	0.045	6.506	0.199	0.375
H3	Empathy → PA	0.225	0.226	0.041	5.533	0.141	0.301
H4	Empathy → PEB	0.348	0.349	0.037	9.288	0.270	0.417
H5	PA → PEB	0.288	0.288	0.040	7.212	0.208	0.364
H6	LP → PEB	0.282	0.283	0.034	8.221	0.214	0.346

All confidence intervals are bias-corrected (BC). All paths are significant at *p* < 0.001.

#### 4.2.2 Mediation analysis

The mediating roles of empathy and place attachment were examined in this study (Table 5). The variance accounted for (VAF) approach was applied to determine the type of mediation, following the guidelines of Hair et al. (2021) and Nitzl et al. (2016). The results showed that for the path from landscape perception to pro-environmental behavior via empathy, the indirect effect was significant [β = 0.122, Standard Deviation *(STDEV)* = 0.020, *t* = 6.218, *p* < 0.001], while the direct effect remained significant (β = 0.282, *p* < 0.001). The VAF value was 30.20%, indicating a partial mediation effect. For the path from landscape perception to pro-environmental behavior via place attachment, the indirect effect was also significant (β = 0.084, *STDEV* = 0.017, *t* = 4.838, *p* < 0.001), and the direct effect remained significant (β = 0.282, *p* < 0.001). The VAF value was 22.95%, indicating partial mediation.

**Table 5:** Mediation analysis results and variance accounted for (VAF).

Path	Original sample (β)	STDEV	*t*-value	Indirect effect	Direct effect	Total effect	VAF (%)
LP → Empathy → PEB	0.122	0.020	6.218	0.122	0.282	0.404	30.20
LP → PA → PEB	0.084	0.017	4.838	0.084	0.282	0.366	22.95

*p* < 0.05 indicates a significant mediating role. VAF ≥ 20% indicates partial mediation; VAF <20% indicates no mediation.

These findings indicate that landscape perception is associated with pro-environmental behavior both directly and indirectly through affective mechanisms. In particular, empathy reflects an immediate emotional response to environmental experience, which has been shown to be closely related tpro-environmental behavior (Ienna et al., 2022). This supports the stimulus–organism–response (SOR) framework, in which environmental stimuli are linked to behavioral outcomes through internal affective processes. By contrast, place attachment represents a more stable emotional bond that develops through accumulated experiences and place-based meaning (Ramkissoon et al., 2013). The relatively stronger mediating role of empathy indicates that immediate emotional responses may play a more prominent role than longer-term attachment in explaining pro-environmental behavior.

## 5 Discussion

### 5.1 Theoretical implications

This study examined the direct and indirect relationships among landscape perception, empathy, place attachment, and pro-environmental behavior. The findings provide important theoretical insights into the affective mechanisms linking environmental perception and behavioral responses. Previous studies have typically conceptualized the “organism” component in the SOR framework as a relatively unified emotional response, often focusing on single affective constructs such as place attachment (Halpenny, 2010; Raymond et al., 2010). By contrast, the present study shows that the organism component is better understood as a differentiated affective structure, in which empathy represents a more immediate response and place attachment reflects a more stable emotional bond. In this sense, the study not only supports prior work on the importance of affect in environmental experience (Brinck, 2018), but also extends SOR theory by deepening the affective dimension of the organism component.

The findings also confirm that landscape perception is significantly associated with both empathy and place attachment, which is consistent with prior research highlighting the role of environmental stimuli in shaping emotional responses (Wartmann et al., 2021a). However, unlike earlier studies that focused primarily on single sensory or aesthetic dimensions (Ren and Kang, 2015), this study conceptualizes landscape perception as a multidimensional construct integrating sensory, aesthetic, and cultural aspects. This extends existing research by showing that a more integrated perception of landscape is associated with both immediate emotional resonance and longer-term place-based attachment. More importantly, this study provides empirical evidence for empathy as a key affective mechanism within landscape experience. While previous studies have mainly discussed empathy as a driver of pro-environmental concern (Berenguer, 2007), its role in spatial and environmental contexts has remained underexplored. This finding also differs from prior studies showing that place attachment may enhance empathy with nature and thereby relate to pro-environmental behavior (Chen et al., 2024). In contrast, the present study indicates that empathy may precede and contribute to the development of place attachment. This suggests that the relationship between empathy and place attachment may be dynamic rather than strictly unidirectional.

Building on this, the results further indicate that empathy exhibits a relatively stronger mediating role than place attachment. This differs from earlier research that has predominantly positioned place attachment as the central affective pathway linking environmental experience and pro-environmental behavior (Junot et al., 2018; Scannell and Gifford, 2010). The present findings suggest that immediate emotional responses may play a more prominent role than longer-term attachment in shaping pro-environmental behavior, indicating the need to distinguish between different temporal layers of affective processes.

### 5.2 Practical implications

**Figure 3:**
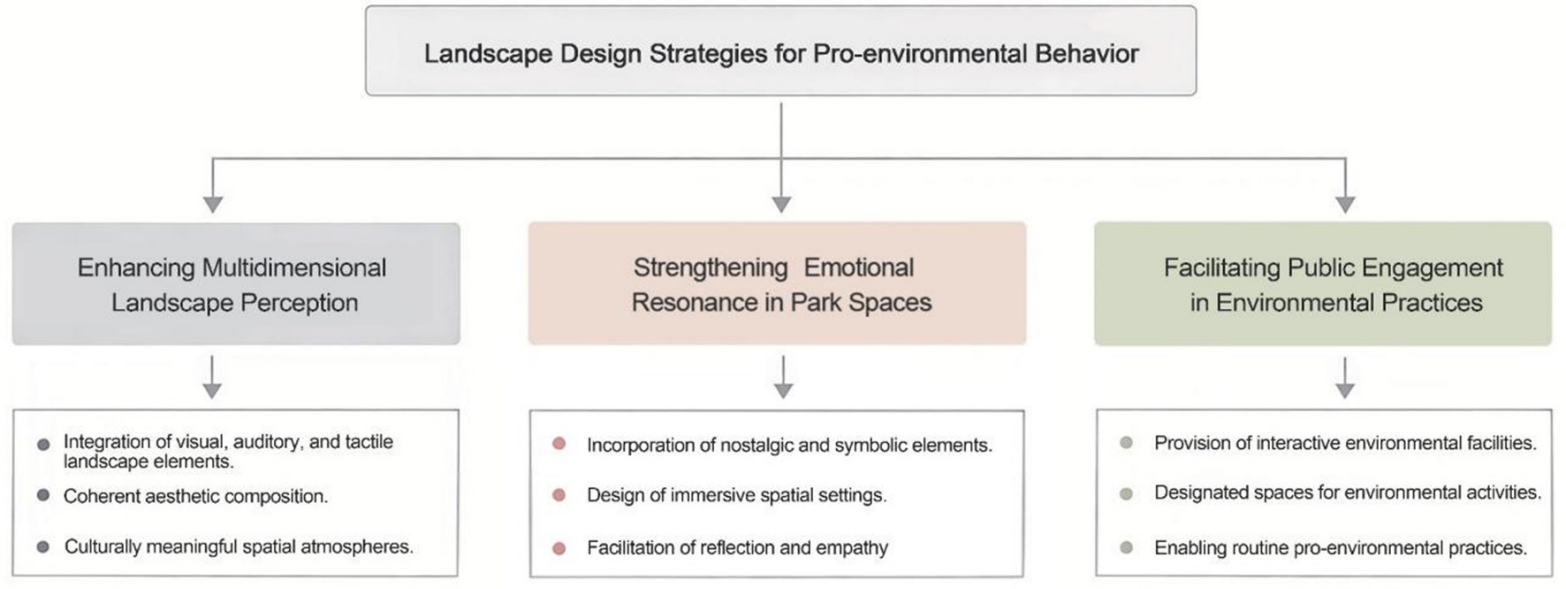
Landscape design strategies for pro-environmental behavior.

The findings of this study provide several practical implications for urban park design and planning aimed at promoting pro-environmental behavior. Figure 3 presents a summary of three complementary design strategies derived from the results, including the enhancement of multidimensional landscape perception, the integration of affective cues, and the facilitation of public participation in environmental practices. From a design perspective, urban parks should be planned to enhance multidimensional landscape perception by integrating visual, auditory, and tactile elements within a coherent spatial composition. Such integration can strengthen users' overall perceptual experience and support both immediate emotional responses and the gradual development of place attachment. This may be achieved through vegetation diversity, natural soundscapes, and carefully designed spatial sequences that create immersive environmental experiences. Emotionally, landscape design should incorporate affective cues that can evoke empathy and emotional resonance. Elements such as symbolic cultural features, narrative landscapes, and reflective spaces can encourage users to form deeper emotional connections with the environment. These interventions are particularly important given the role of empathy as an immediate affective mechanism associated with pro-environmental behavior. At the level of public participation, urban parks should provide spatial opportunities that translate emotional engagement into behavioral responses. This can be supported through interactive facilities, environmental education installations, and designated areas for ecological activities, which enable users to actively engage in pro-environmental practices within the park setting. Overall, these strategies highlight the importance of designing urban parks not only as physical environments but also as affective and behavioral spaces, where perceptual experience and emotional processes are closely associated with environmentally responsible actions.

### 5.3 Limitations and future research

Although this study examined the relationships among landscape perception, empathy, and place attachment in relation to pro-environmental behavior within the SOR framework using data from three representative urban parks, several limitations should be acknowledged. First, the study relied primarily on self-report measures, which may introduce response bias and common method variance. Future research could triangulate subjective evaluations with objective indicators of park environments, such as vegetation structure, canopy coverage, soundscape characteristics, microclimatic conditions, and the spatial distribution of facilities, measured through Geographic Information System (GIS)-based analyses and systematic field observations. Second, because the empirical evidence was drawn from urban parks in a specific cultural and geographical context, the generalizability of the findings to other settings remains to be verified. Cross-regional and cross-cultural comparative studies, for example between Eastern and Western urban parks with different design logics and experiential patterns, would help strengthen external validity. Third, to further validate subjective psychological measures and capture immediate affective responses during landscape interaction, future studies could incorporate physiological indicators, such as heart-rate variability, skin conductance, or body temperature collected via wearable devices or mobile sensors (Purohit et al., 2020). These measures may improve the precision of emotional assessment and provide deeper insight into how empathy functions in actual park environments. Finally, extending the analytical framework to include additional constructs, such as environmental values or social capital, may help clarify their potential mediating or moderating roles in the relationships among the studied variables.

## 6 Conclusion

This study developed and tested an integrated model within the SOR framework that incorporates multidimensional landscape perception, immediate empathy, and enduring place attachment. The results indicate that landscape perception is associated with pro-environmental behavior both directly and indirectly through emotional pathways, with empathy exhibiting a stronger mediating role than place attachment. These findings highlight the layered nature of emotional processes in human–environment interactions, in which short-term affective resonance and long-term emotional bonds operate as distinct but complementary mechanisms. By introducing empathy as a cross-disciplinary construct into landscape research, this study extends the conceptualization of the “organism” component in SOR theory and provides empirical evidence clarifying the relationship between empathy and place attachment in shaping pro-environmental behavior. At the practical level, the findings underscore the importance of integrating emotional dimensions into urban planning and landscape design. Beyond functional performance, public spaces should foster affective resonance and place attachment through diversified landscape features, aesthetic atmospheres, and culturally meaningful elements, thereby strengthening users' sense of environmental responsibility. This perspective aligns with the principles outlined by the World Health Organization (2017), which emphasize enhancing the social and health benefits of urban green spaces through design quality, social engagement, and sustained use and protection. Overall, this study advances understanding of affective mechanisms associated with pro-environmental behavior and offers design-oriented insights to support the sustainable development and long-term management of urban green spaces.

## Data Availability

The raw data supporting the conclusions of this article will be made available by the author, without undue reservation.
